# Supplementary material for: Anomalous flocking in nonpolar granular Brownian vibrators
Source: Nat Commun. 2024 Jul 17;15:6032. doi: 10.1038/s41467-024-50479-7 (PMC11255308; doi:10.1038/s41467-024-50479-7)
Supplement: Supplementary file 1 — Supplementary Information [file 41467_2024_50479_MOESM1_ESM.pdf]

Supplementary Information of the "Anomalous flocking in nonpolar granular Brownian vibrators"

Yangrui Chen[1] and Jie Zhang[1,2*]

[1*]School of Physics and Astronomy, Shanghai Jiao Tong University, Shanghai 200240, China.
[2*]Institute of Natural Sciences, Shanghai Jiao Tong University, Shanghai 200240, China.

*Corresponding author(s). E-mail(s): jiezhang2012@sjtu.edu.cn;

In the Supplemental Information, we provide more detailed descriptions of the following:

1. Supplementary Methods
(1.1) The dynamics of active particles.
(1.2) Voronoi diagrams at various values of ϕ.
(1.3) Two-dimensional (2d) maps showing the absolute value of the hexatic order parameter, $\left|\psi_6^j\right|$, at different ϕ values. And the global mean hexatic order and 2d correlations of the hexatic order parameter, ψ_6^j.
(1.4) The probability distribution functions (PDFs) of the particle displacement in the granular fluid, collective fluid, poly-crystal, and crystal, at different ϕ values.
(1.5) The average curl of the particle displacement field.
2. Supplementary Discussion
(2.1) Self intermediate scattering functions $F_s(q,t)$.
(2.2) Important timescales observed in the experiment.
(2.3) The spatial connected correlation function of the displacements
(2.4) The pair correlation function(radial distribution function) $g(r)$.
3. Supplementary References

1 Supplementary Methods

1.1 The dynamics of active particles

From the theoretical work by Caprini et al.[1], the dynamics of active particles reads:

$$m\dot{\mathbf{v}}_\mathbf{i} = -\gamma \mathbf{v}_\mathbf{i} + \mathbf{F}_\mathbf{i} + \gamma_0 v_0 \mathbf{n}_\mathbf{i} + \sqrt{2\Lambda\gamma}\eta_\mathbf{i}, \tag{1}$$

$$\dot{\theta}_\mathbf{i} = \sqrt{2D_r}\mathbf{\Theta}_\mathbf{i} \tag{2}$$

Here the effective dissipative force $|\mathbf{F_d}| \equiv mv_0/\tau_I$. When the system reaches a steady state, the rate of energy dissipation within the system equals the rate of energy input from the active force. Therefore, the magnitude of the active force $|\mathbf{F_a}| = |\mathbf{F_d}| \equiv mv_0/\tau_I = \gamma_0 v_0$, where $\gamma_0 = m/\tau_I$ is the effective damping coefficient.

Our analysis focuses on the comparison of the dimensionless coefficients between the interparticle force $\mathbf{F_i}$ and the active force $\gamma_0 v_0 \mathbf{n_i}$. First, by calculating the distribution of particle number density, we can determine the equivalent interaction force $\mathbf{F_i}$ of inelastic collisions between particles.

The radial distribution function, also known as the pair correlation function, is defined as:

$$g(r) = \frac{L^2}{2\pi r \Delta r N(N-1)} \sum_{j\neq k} \delta(r - |\vec{r}_{jk}|), \tag{3}$$

where L is the system size, N is the total number of particles, $|\vec{r}_{jk}|$ refers to the relative distance between two particles j and k, and Δr is a small increment along the radial direction.

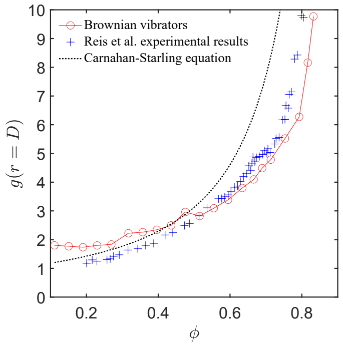

Supplementary Fig. 1 The first peak of the pair correlation function in three systems. The figure of $g(r = D)$ versus ϕ. Red circles refer to our experimental results of Brownian vibrators. Blue pluses are the experimental results with vibrated stainless steel spheres in Ref.[2]. The black dotted line is the Carnahan-Starling equation.

The particle configurations of our system are different from those of equilibrium hard disks and the previous experiment using vibrated stainless steel spheres Ref.[2], as can be seen from the curve of $g(r = D)(\phi) \equiv g_D(\phi)$ shown in Supplementary Fig. 1 with D being the particle diameter. The black dotted line refers to the theoretical curve of Carnahan-Starling equation $g_D^{CS}(\phi) = [1 - 7/16\phi]/(1 - \phi)^2$, which is derived from the equation of state for non-attracting hard spheres[3].

Assuming that the inelastic collisions between particles can be effectively represented by an interparticle attractive potential, the radial distribution function is given by $g(r) = \exp(-V(r)/(kT)) \cdot g_{\mathrm{eq}}(r)$, where $V(r)$ represents the effective potential and $kT \equiv \frac{1}{2}mv_T^2$ can be estimated from the thermal velocity of the particles, denoted as v_T. The definition of the thermal velocity is provided below. The equilibrium radial distribution function, $g_{\mathrm{eq}}(r)$, for purely repulsive hard spheres is obtained from the BGY theory as described in Ref. [4]. The interparticle force is given by $F_i = -\frac{d(V(r))}{dr}$, and its maximum value is determined by:

$$F_{\max} = \frac{1}{2}mv_T^2 \left[\frac{d \ln \left(\frac{g(r)}{g_{\mathrm{eq}}(r)} \right)}{dr} \right]_{\max} . \tag{4}$$

The thermal velocity, v_T, can be obtained by calculating the ensemble-averaged root mean square (RMS) velocity across different packing fractions, ϕ, using the formula:

$$v_T = \sqrt{\frac{\sum_{i=1}^{N_\phi} v_i^2}{N_\phi}}, \tag{5}$$

where N_ϕ represents the total number of particles at a given packing fraction ϕ, and v_i denotes the RMS velocity of a specific particle i. Here the thermal velocity $v_T = 1.4 \pm 0.1 D \cdot s^{-1}$ is weakly affected by ϕ [5].

Thus, we have completed the calculation of the equivalent attractive force of inelastic collisions at different packing fractions. Next, we will introduce the calculation details of the Péclet number Pe at different packing fractions.

In our experiment, the persistence time, denoted as τ_p, can be determined from the cutoff time corresponding to the initial segment of superdiffusion. This segment is identified by the condition where the local slope of the mean square displacement (MSD) curve, represented by $k^* = \frac{d(\log(MSD))}{d(\log(t))}$ in double logarithmic coordinates, is greater than 1, as illustrated in Supplementary Fig. 2. Considering the fluctuations of the measured slope k^* in the experiment, where $k^* = 1 \pm 0.1$ within the time scale of the random walk, we can determine τ_p as $\tau_p = \max(t(k^* > 1.1))$. This maximum value of t is depicted in Supplementary Fig. 3.

To determine the root mean square of the active velocity, denoted as v_0, single-particle experiments are conducted. The estimation of v_0 is obtained as follows:

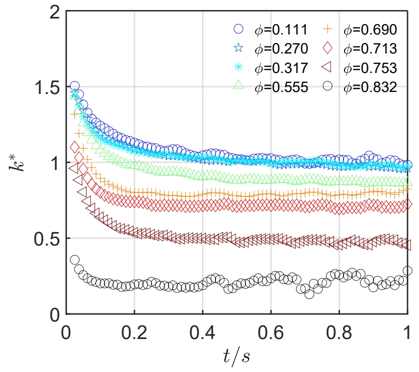

Supplementary Fig. 2 The local slope of the mean square displacement curve. The local slope of the mean square displacement (MSD) curve in double logarithmic coordinate $k^* = \frac{d(log(MSD))}{d(log(t))}$.

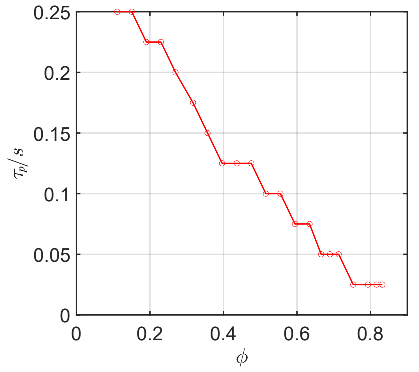

Supplementary Fig. 3 The persistence time τ_p. The stair-like structure is due to the finite time resolution of 0.025 s. Since the experimental frame rate is 40 frames per second, the time resolution is 0.025s.

$$v_0 = \sqrt{\langle \overline{\mathbf{v}^2(t)} \rangle} \approx 1.52\mathrm{D} \cdot \mathrm{s}^{-1} \approx 24.3\mathrm{mm} \cdot \mathrm{s}^{-1} \tag{6}$$

Here, the notation $\langle \cdots \rangle$ represents the average over repeated experiments, and $\overline{\cdots}$ indicates the time average over the particle trajectory.

The persistence length of particles, denoted as $v_0\tau_p$, quantifies the distance over which a particle maintains directional persistence. It represents the range within which its motion remains coherent before being randomized by random forces. And the Péclet number $Pe \equiv v_0/(D_r D)$ is viewed as the ratio between persistence length and particle size which in this case is D=16 mm. Here the persistence time $\tau_p = 1/D_r$ [1]. Therefore,

theoretically, Pe can also be calculated through the definition. However, due to the higher spatial resolution precision in the experiment (0.29mm per pixel) compared to the temporal resolution precision (average particle displacement of 0.6mm within 0.25 seconds), the Pe presented in the main text is obtained from the corresponding mean square displacements over the persistent time.

Consider the motion of an individual Brownian particle on an aluminum alloy plate. When the vibrational driving is stopped, the time required for the particle with an average velocity v_0 to decelerate to 0 due to friction is $\tau_f = v_0/(\mu g)$, where μ is the friction coefficient and g is the acceleration due to gravity. μ has been measured to be approximately 0.420. This measurement was obtained by determining the angle of repose through an experimental setup involving a gradual and continuous tilting of a slope from its horizontal position. The angle of repose is identified as the point where the particle exhibits incipient instability and starts to slide. Then we obtain:

$$\tau_f = \frac{v_0}{\mu g} \approx 5.91\text{ms}. \tag{7}$$

In our vibration experiments, the motion of particles can be classified into two types: (a) collisions with the vibrating platform, and (b) a projectile-like leap motion where particles momentarily detach from the platform. The inertia time, denoted as τ_I, of an individual vibrating particle is determined by two factors: the time required for the velocity to be completely dissipated through multiple collisions with the platform, and the cumulative duration of all the leap periods between these collisions. Mathematically, it can be expressed as:

$$\tau_I = \frac{\tau_f}{\tau_c}(\tau_c + \tau_h). \tag{8}$$

Here, τ_c is the contact time for mutual collisions of spheres with radius R, and $\tau_h = 38ms$ denotes the average duration of a single particle's leap, which is measured using a high-speed camera. The contact time τ_c can be calculated using a formula derived from the reference [6–8]:

$$\tau_c = 3.218R\frac{[\sqrt{2}\pi\rho(1-\nu^2)]^{2/5}}{Y^{2/5}v_c^{1/5}} \tag{9}$$

In our experiment, a single collision between our Brownian vibrators and the platform can be effectively approximated as a collision between one of the 12 inclined legs and the platform. Considering that the legs of the particles are cylindrical with a radius of 0.5mm, we can treat the collision as a mutual collision between spheres with a radius R=0.5mm for calculation purposes.

Our Brownian vibrators are 3d-printed using CBY-01 resin. JS-UV-CBY-01 Light Yellow High-Temperature Hard Material is an ABS-Like Stereoscopic Modeling Resin with precise and durable properties, which is used in solid-state laser light curing molding. At a temperature of 25 °C, the density$\rho = 1.14\text{g}\cdot(\text{mm})^{-3}$, Poisson's ratio:

$\nu = 0.39$, Young's modulus: $Y = 2\text{GPa}$, the viscosity is 400 mPa $\cdot$ s. In our 3d-printing process, we utilized a curing depth of 0.135 mm and a construction layer thickness of 0.05 mm. The collision velocity, denoted as v_c, can be estimated as $v_c = \frac{\tau_h g}{2} \approx 186\text{mm}\cdot\text{s}^{-1}$, where τ_h is the average duration of a single particle's leap and g represents the acceleration due to gravity. The maximum velocity of the vibrating platform along the z-axis is $46.8\text{mm}\cdot\text{s}^{-1}$. Since the collision velocity term v_c in the equation above is raised to the power of 1/5, the correction for the platform motion's impact on the collision velocity can be neglected in the calculation.

By substituting the above parameters into equation (A9), we obtain $\tau_c \approx 0.193\text{ms}$. Then, by plugging τ_c into equation (A8), we get $\tau_I \approx 1.17\text{s}$.

The magnitude of the equivalent dissipative force is given by $|\mathbf{F_d}| \equiv mv_0/\tau_I$. After reaching a steady state, the energy dissipated by the particle is equal to the energy input from the active force. Therefore, the magnitude of the active force $|\mathbf{F_a}| = |\mathbf{F_d}| \equiv mv_0/\tau_I = \gamma_0 v_0$, where γ_0 is the equivalent drag factor, given by m/τ_I, and m is the mass of the particle.

Here the magnitude of active force $\gamma_0 v_0$ is generated through the friction between the particle and the platform due to the unique structure of the inclined legs of the particles, which is different with the simulation[1]. However, this distinction does not significantly impact the relevant analysis presented in the main text, as it primarily involves comparing the dimensionless factors associated with the second and third terms.

By comparing the maximum equivalent attractive force and the active force, the critical value can be evaluated.

$$Pe_c = F_{max}/(\gamma v_0) \cdot Pe = \frac{1}{2} m v_T^2 \left[\frac{d \ln \left(\frac{g(r)}{g_{eq}(r)} \right)}{dr} \right]_{max} /(\gamma v_0) \cdot Pe \equiv \lambda Pe \tag{10}$$

The dimensionless coefficient, denoted as λ, exhibits an expected increase with an increase in ϕ. This trend is consistent with the fact that the effective attraction force also increases with ϕ due to the increasing collision frequency. For instance, at $\phi = 0.270$, λ is approximately 0.72, while at $\phi = 0.317$, λ reaches around 1.04. These values indicate that when the maximum effective attractive force surpasses the active forces, collective behavior starts to emerge according the theoretical predictions[1].

1.2 Voronoi diagrams

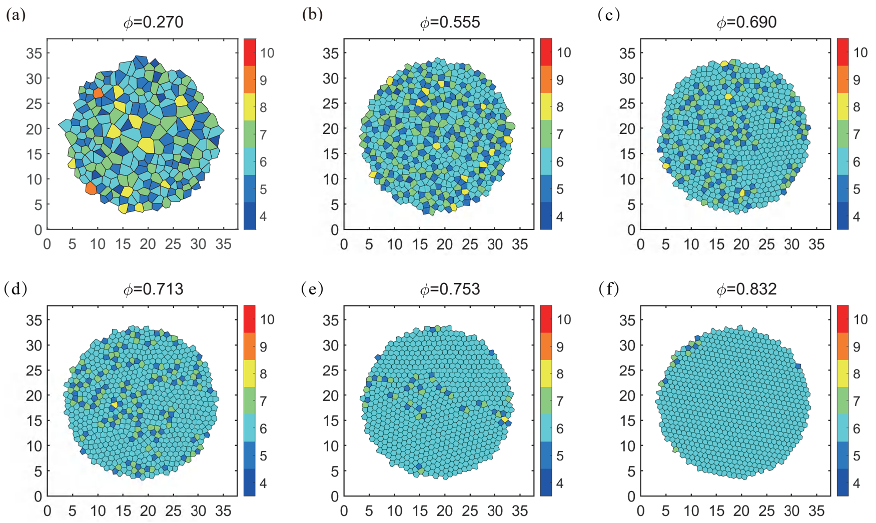

Supplementary Fig. 4 The Voronoi diagrams. (a-f) Voronoi diagrams at various $\phi's$. The color bar represents the order of the Voronoi cells.

Supplementary Fig. 4 illustrates the Voronoi diagrams at different values of ϕ. In the granular fluid phase ($\phi = 0.270$), the Voronoi cell order, represented by the number of edges, ranges from 4 to 9 and exhibits a random spatial distribution. In the flocking fluid phase ($\phi = 0.555$), the order ranges from 4 to 8, and there is no noticeable localized ortho-hexagonal aggregation. In comparison, in the poly-crystalline phase ($\phi = 0.713$), the majority of Voronoi cells are of order six, and the grain boundaries consist of pairs of grains with orders 5 and 7. This observation holds true for $\phi = 0.753$ as well. With the exception of defects near boundaries, the crystalline region at $\phi = 0.832$ comprises Voronoi cells of order six that span the entire system. By utilizing the area S_v of Voronoi polygons, we can calculate the local packing fraction $\phi_{local} = \frac{\pi D^2}{4S_v}$ and analyze its distribution and fluctuation.

1.3 The hexatic order parameter

The hexatic order parameter, ψ_6^j, quantifies the similarity between the structure surrounding a particle j and a perfect hexagonal arrangement. It is defined as $\psi_6^j = \frac{1}{n_j}\sum_{k=1}^{n_j} e^{i6\theta_{jk}}$, where n_j represents the number of nearest neighbors of particle j, and θ_{jk} denotes the angle between the vector $\vec{r}_{jk} \equiv \vec{r}_k - \vec{r}_j$ and the x-axis. Notably, n_j is not restricted to six; it can take different values, such as 2 for a linear structure, 3 for a trident structure, 4 for a center particle with four neighbors, and so on.

To illustrate, let's consider the case where $n_j = 2$. When the center particle and its two neighbors form a straight line, ψ_6^j equals 1. Consequently, ψ_6^j characterizes the local particle-scale symmetry.

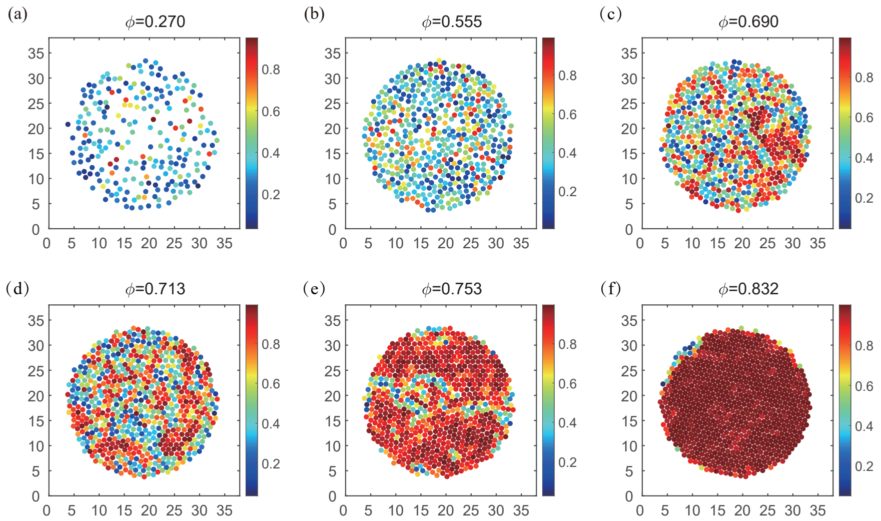

Supplementary Fig. 5 The hexatic order parameter. (a-f) The absolute value of hexatic order parameter $\left|\psi_6^j\right|$ at various $\phi' s$. The color bar represents the absolute value of hexatic order parameter.

The 2d maps displaying the absolute value of the hexatic order parameter, $\left|\psi_6^j\right|$, at various ϕ values are presented in Supplementary Fig. 5. Each panel in the figure corresponds to the same particle configurations as shown in Supplementary Fig. 4.

The global mean hexatic order parameter, $\psi_6^{global} = \langle|\frac{1}{N}\sum_{j=1}^{N}\psi_6^j|\rangle$, is calculated by averaging the absolute value of the hexatic order parameter for all particles over different configurations. Here, N represents the total number of particles, and $\langle ... \rangle$ denotes the average over different particle configurations.

In Supplementary Fig. 6, we compare the global mean ψ_6^{global} observed in our experiment with the results from Ref.[2], particularly showing a plateau for packing fractions $\phi \leq 0.436$.

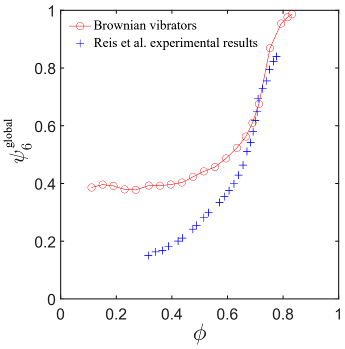

Supplementary Fig. 6 The global mean of ψ_6 at various $\phi's$. Red circles refer to Brownian vibrators. Blue pluses are the results of Ref.[2].

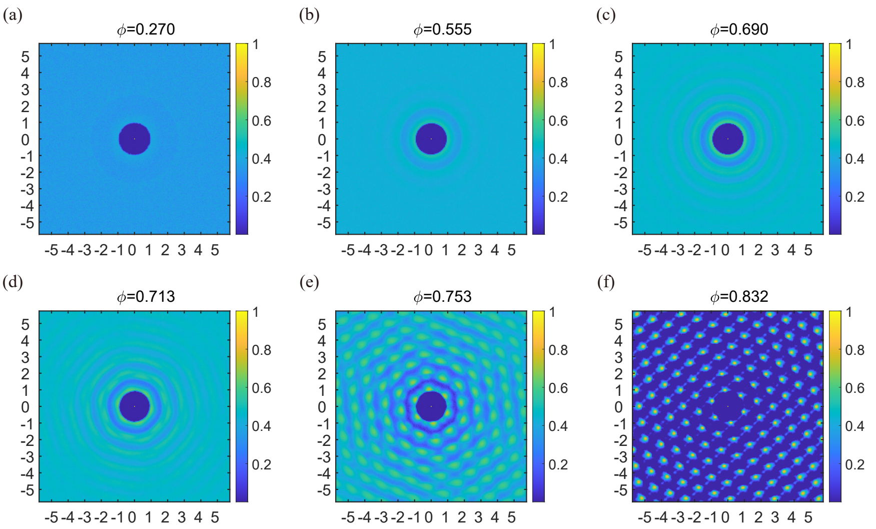

Supplementary Fig. 7 The 2d correlations of the local hexatic order. (a-f) The 2d correlations of the local hexatic order $g_6^{2D}(r,\theta)$ at various $\phi's$.

Moving on to Supplementary Fig. 7, we present the two-dimensional correlations of the local hexatic order, denoted as $g_6^{2D}(r,\theta) = \frac{L^2}{r\Delta r\Delta\theta N(N-1)} \sum_{j\neq k} \delta(r - |\vec{r}_{jk}|)\delta(\theta - |\theta_{jk}|)\psi_6^j\psi_6^k$. It is evident from the figure that $g_6^{2D}(r,\theta)$ exhibits isotropic behavior for $\phi \leq 0.690$, while it demonstrates hexagonal symmetry for $\phi \geq 0.713$, indicating clear crystalline characteristics.

1.4 The PDFs of particle displacement

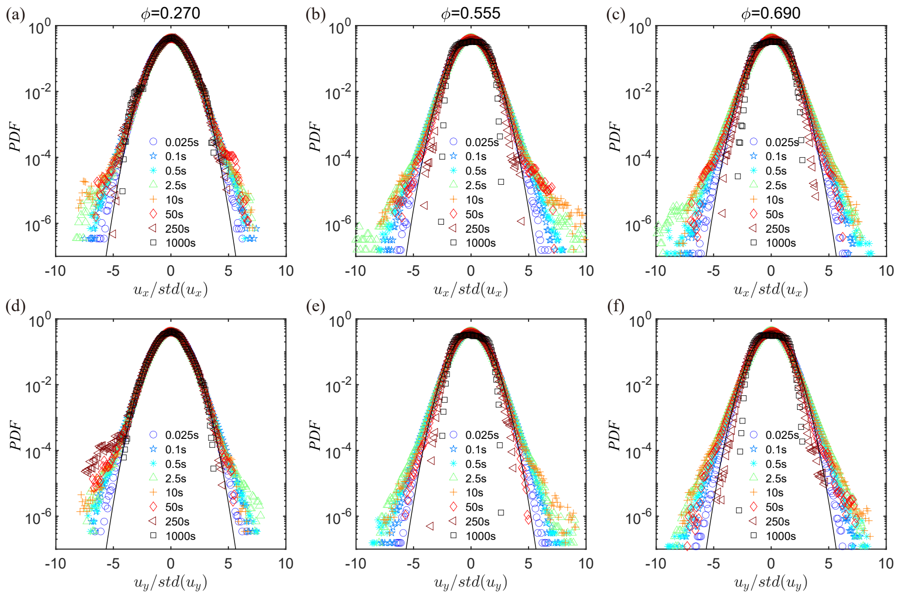

Supplementary Fig. 8 The PDFs of particle displacement at $\phi = 0.270, 0.555, 0.690$. The PDFs of $u_\alpha/std(u_\alpha)$ (α refers to the x or y component) at the volume fractions of $\phi = 0.270, 0.555, 0.690$. Different colors and markers represent various time intervals. The black solid lines refer to Gaussian distributions. (a-c) $u_x/std(u_x)$ (d-f)$u_y/std(u_y)$.

The probability density functions (PDFs) of u_x and u_y, representing the x and y components of the particle displacement vector $\vec{u}$, are presented in Supplementary Fig. 8 and Supplementary Fig. 9, respectively. In these figures, the horizontal coordinates are normalized by the corresponding standard deviation of particle displacement. The solid black lines in the figures depict Gaussian distributions for comparison.

Notably, none of the PDF curves exhibit a Gaussian shape. As $u_x/std(u_x)$ or $u_y/std(u_y)$ varies above and below zero, each curve progressively deviates from the Gaussian distribution. However, in most cases, except for $\phi = 0.753$, the Gaussian distributions can still offer an approximate description of the PDFs, particularly for the central part of the distributions. It is primarily in the tails of the PDFs, where the probabilities are less than 10^{-3}, that the Gaussian approximation becomes less accurate.

In the case of the volume fraction $\phi = 0.753$, the PDFs of $u_x/std(u_x)$ and $u_y/std(u_y)$ exhibit the most significant deviations from Gaussian behavior at long time intervals. This deviation is attributed to the coexistence of a large number of nearly perfect crystal particles and a relatively small number of grain boundary particles, which leads to distinct features in the PDF distributions.

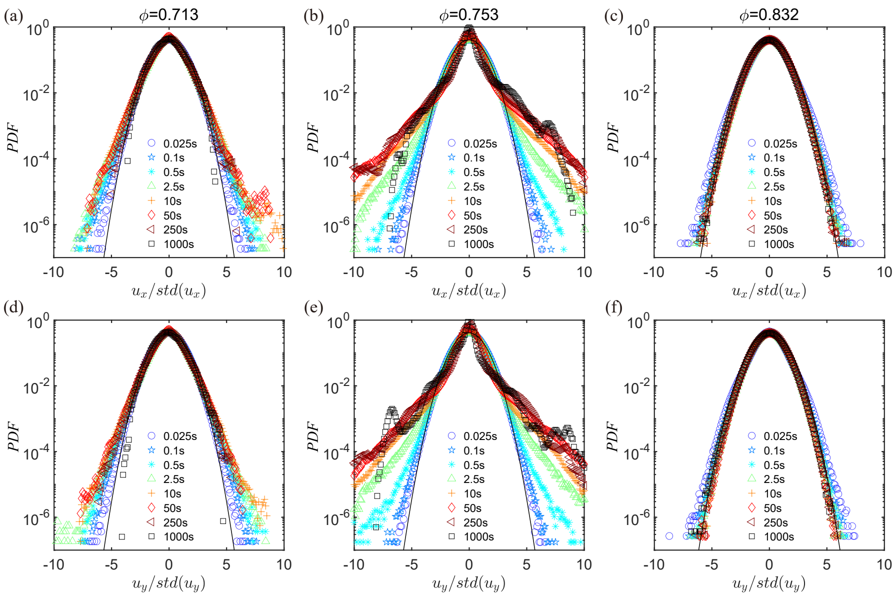

Supplementary Fig. 9 The PDFs of particle displacement at $\phi = 0.713, 0.753, 0.832$. The PDFs of $u_\alpha/std(u_\alpha)$ (α refers to the x or y component) at the volume fractions of $\phi = 0.713, 0.753, 0.832$. Different colors and markers represent various time intervals. The black solid lines refer to Gaussian distributions. (a-c) $u_x/std(u_x)$ (d-f)$u_y/std(u_y)$.

The departure of the PDFs from Gaussian behavior can be quantitatively assessed using the kurtosis, as depicted in Supplementary Fig. 10. The kurtosis is defined as follows:

$$\frac{\mu_4}{\sigma^4} = \frac{\frac{1}{n}\sum_{i=1}^{n}(u_i - \bar{u})^4}{(\frac{1}{n}\sum_{i=1}^{n}(u_i - \bar{u})^2)^2}, \tag{11}$$

where u_i represents a random variable, which can refer to either $u_x/std(u_x)$ or $u_y/std(u_y)$. It is important to note that the kurtosis of a Gaussian distribution equals 3.

1.5 The average curl of the particle displacement field

We focus on the particle displacement field to quantify the large-scale collective motion. First, the particle density field can be coarse-grained with the kernel function $\Psi(\vec{r}) = e^{-(2\vec{r}/D)^2}$:

$$\rho(\vec{r}) = \sum_{i=1}^{N} \Psi(\vec{r} - \vec{r}_i). \tag{12}$$

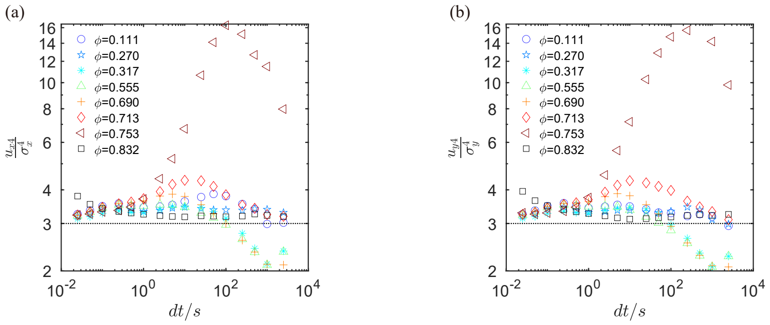

Supplementary Fig. 10 The kurtosis of the PDFs of particle displacement. The kurtosis of the PDFs of $u_x/std(u_x)$ (a) and $u_y/std(u_y)$ (b) for a series of time intervals and different packing fractions $\phi's$.

Next, the particle displacement field can be coarse-grained within a time interval Δt:

$$\vec{u}(\vec{r}, \Delta t) = \rho(\vec{r})^{-1} \sum_{i=1}^{N} \vec{u}_i(\Delta t)\Psi(\vec{r}). \tag{13}$$

Here, the displacement of the particle i is $\vec{u}_i(\Delta t) = (\vec{r}_i(t_0 + \Delta t) - \vec{r}_i(t_0))$, and the collective behavior is evident in Fig. 1 (e) in the main text for $\Delta t = 100s$. In a quasi-two-dimensional system, the average curl of the displacement field Ω is defined as:

$$\begin{aligned} \Omega &= \langle [\nabla \times \vec{u}(\vec{r})]_z \rangle \\ &= \langle \rho(\vec{r})^{-2} \sum_{i=1}^{N} \sum_{j=1}^{N} \Psi(\vec{r})_j \left[\nabla \Psi(\vec{r})_i \times \vec{u}_{ij}\right]_z \rangle. \end{aligned} \tag{14}$$

Here, the angle brackets represent the spatial average followed by a time average over different t_0.

2 Supplementary Discussion

2.1 Self intermediate scattering functions $F_s(q,t)$

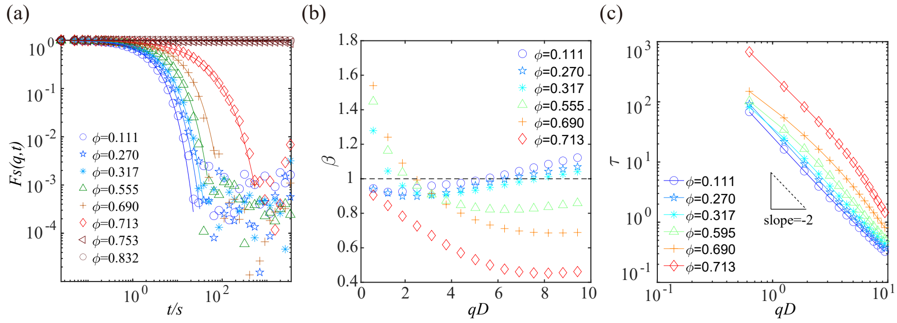

Supplementary Fig. 11 Self intermediate scattering functions. (a) Self intermediate scattering functions $F_s(q,t)$ with $q=\pi/D$. The solid lines are fitting $F_s(q,t)=exp(-(t/\tau)^{\beta})$. (b-c) The β and τ versus q.

The intermediate scattering function $F_s(q,t)$ characterizes structural relaxation for a given wavevector $\vec{q}$:

$$F_s(q,t) = \frac{1}{N}\Sigma_{j=1}\langle e^{-i\vec{q}\cdot(\vec{r}_j(t)-\vec{r}_j(0))}\rangle$$
$$= \frac{1}{N}\Sigma_{j=1}e^{-\frac{1}{4}q^2\langle(\vec{r}_j(t)-\vec{r}_j(0))^2\rangle}, \quad (15)$$

where $r_j(t)$ refers to the trajectory of the particle j at time t, N is the total particle number, and the average $\langle\rangle$ is over all possible initial configurations $\vec{r}_j(0)$. The second equality assumes Gaussian $\vec{r}_j(t)-\vec{r}_j(0)$ [9], approximately valid, as shown in Supplementary Fig. 8, Supplementary Fig. 9 and Supplementary Fig. 10.

Supplementary Fig. 11(a) shows the $F_s(q,t)$ with $q=\pi/D$, corresponding to the scale of $2D$. $F_s(q,t)$ decay monotonically with an increasing relaxation time with ϕ, except at $\phi=0.832$. We fit $F_s(q,t)$ with an stretched exponential $F_s(q,t) = exp(-(t/\tau)^{\beta})$ and plot β and τ for different q in Supplementary Fig. 11(b-c), respectively. There are three branches of $\beta(q)$ in Supplementary Fig. 11(b), corresponding to the granular fluid, flocking fluid, and poly-crystal. When $\phi=0.111$ and 0.270, $\beta<1$ for small q and increase above one at $q\approx 2\pi/D$. The weak subdiffusion at large scales is consistent with Fig. 1(g) in the main text, reflecting the effect of finite boundaries. The flocking fluid shows more complex ϕ dependent behaviors of $\beta(q)$. For $q<2/D$, corresponding to the scales larger than πD, $\beta(q)>1$ for all ϕ, showing characteristics of the large-scale collective motions. For $q\geq 2/D$, when $\phi=0.317$, β stays slightly below one within $2/D<q<2\pi/D$ and then goes above one for $q>2\pi/D$, similar to the curves of the granular fluid. When $\phi=0.555$ and 0.690, β remain below

one for $q > 2\pi/D$ and reach around 0.7 for $\phi = 0.690$, confirming subdiffusive glassy behaviors shown in Fig. 1(g) in the main text. When $\phi = 0.713$ and 0.753, $\beta(q)$ starts near one and decreases significantly with q, consistent with Fig. 1(g) in the main text, verifying highly constrained grain-boundary particles. According to Eq.(4) and $F_s(q,t) = exp(-(t/\tau)^{\beta})$, $\tau(q) \propto q^{\kappa(q)}, \kappa(q) = -\frac{2}{\beta(q)}$, consistent with Supplementary Fig. 11(c). For example, when $\phi = 0.111$ and 0.270, $\kappa(q) \approx -2$ with a weak dependence on q, whereas when $\phi = 0.713$, $\kappa(q) \approx -2$ for small q and approach -4.4 at high q in Supplementary Fig. 11 (c).

2.2 Important timescales in the experiment

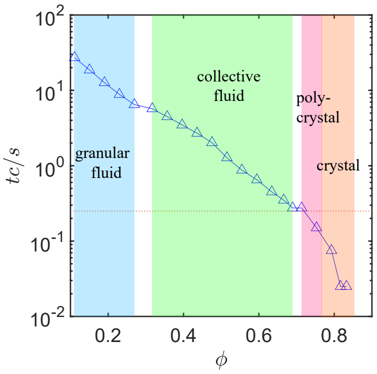

Supplementary Fig. 12 The mean free time of particles. The mean free time of particles was measured at various packing fractions. A red dotted line marks the characteristic time t=0.25s, representing the onset of the single-particle's diffusive motion.

Our experiment involves four crucial timescales:

(1) The time interval of stochastic driving, estimated by the vibration frequency of the electromagnetic shaker, is approximately $1/f = 0.01$ seconds.

(2) The persistence time, denoted as τ_p, is obtained from the particle's mean square displacement (MSD) curve and is around 0.25 seconds for a single particle system.

(3) The particle's mean free time, denoted as t_c, is illustrated in Supplementary Fig. 12. Here, $t_c(\phi)$ is defined as the time when the MSD of particles at a given packing fraction ϕ reaches the mean separation distance of neighboring particles, which is equal to $(\sqrt{\pi/(2\sqrt{3}\phi)} - 1)D$. In the granular fluid phase $(0.111 \le \phi \le 0.270)$ and the flocking fluid phase $(0.317 \le \phi \le 0.690)$, which are the most interesting regimes in terms of physics, the mean free time between particle-particle collisions is much larger than 0.25 seconds. This indicates that stochastic driving randomizes the particle's velocities well before two adjacent particles interact.

(4) The period of collective motion around the system's center is approximately 4000 seconds. In the flocking fluid phases, the MSD peaks around 2000 seconds correspond to half a rotation period of the outermost particles' collective motion. These particles contribute the most to the MSD, as their displacement follows an approximately semicircular path from one side of the system to the other, covering a distance of about 30 particle diameters. Once the displacement reaches this magnitude, the MSD starts to decrease.

2.3 The spatial connected correlation function of the displacements

The spatial correlation function of the instantaneous velocities, as depicted in Supplementary Fig.13(a), demonstrates that the velocity field lacks polar order in both the granular fluid phase and the flocking fluid phase. However, for dt=10s and 100s in Supplementary Fig. 13(c) and (d), the spatial correlation function of the displacements shows long-range spatial correlations at $\phi = 0.555, 0.713$ and 0.832, confirming that there is no collective motion at short time scales, and the flocking fluid phase can only be observed at time scales greater than 10s.

Here the spatial connected correlation function of the displacements is defined as:

$$C(r) = \frac{\sum_{i,j} \delta u_i \cdot \delta u_j \delta(r - r_{ij})}{\sum_{i,j} \delta(r - r_{ij})},$$

δu_i and δu_j are the displacement of the particles at positions i and j in the time interval of dt.

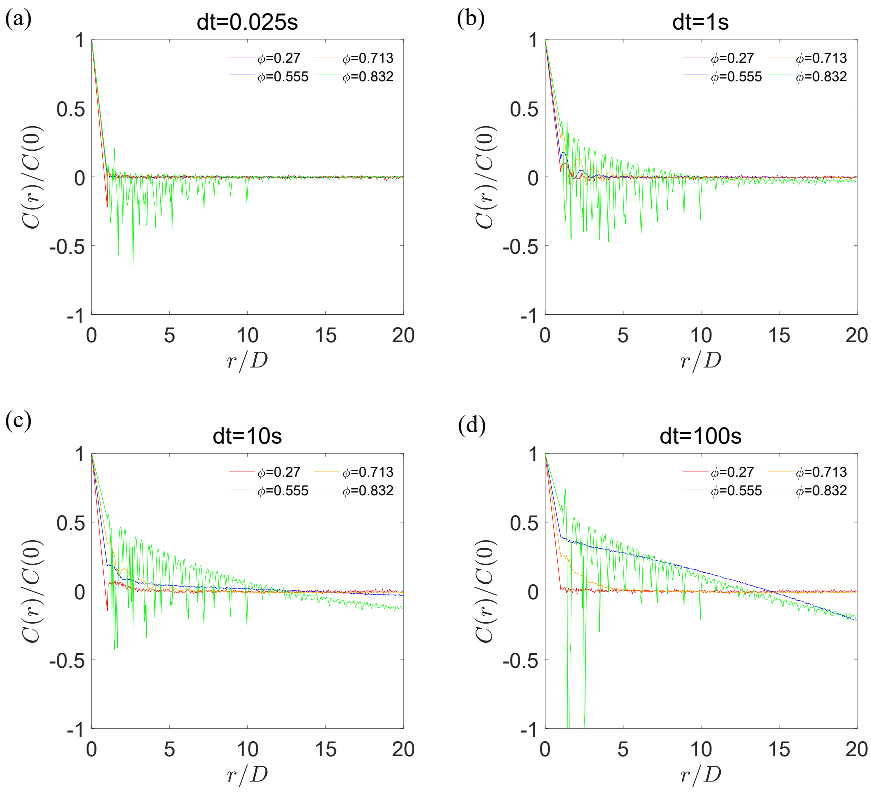

Supplementary Fig. 13 The spatial connected correlation function of the displacements. (a) The time interval dt=0.025s, while it's the same as the connected correlation function of the instantaneous velocities; (b-d) dt=1s,10s,100s.

2.4 The pair correlation function g(r)

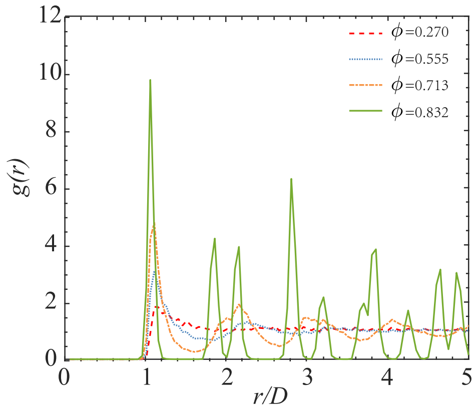

Supplementary Fig. 14 The pair correlation functions. The pair correlation functions $g(r)$ at $\phi = 0.270$(granular fluid phase), $\phi = 0.555$(flocking fluid phase), $\phi = 0.713$(poly-crystal phase) and $\phi = 0.832$(crystal phase).

Supplementary Fig. 14 plots pair correlation functions $g(r)$ of different phases, whose first peak corresponds to the maximum possible center-to-center distance d_1 between neighboring particles. At $\phi = 0.832$, the sharp peaks at $\sqrt{3}d_1$, $2d_1$ and $\sqrt{7}d_1$ indicate an almost perfect crystal. At $\phi = 0.713$, the system forms a poly-crystal, showing a slightly larger value of d_1 on $g(r)$ than that of $\phi = 0.832$. Moreover, the peak at $\sqrt{3}d_1$ symbolizes the triangular lattice. Still, it is almost buried within an extensive shoulder of the peak around $2d_1$ due to grain boundaries. At $\phi = 0.555$, the system forms a flocking fluid, where the first peak shifts further right than that of $\phi = 0.713$. The crystalline feature is washed out as the double peaks near $\sqrt{3}d_1 \sim 2d_1$ disappear entirely and are replaced with a broad single peak, indicating liquid-like structures. Moreover, there are no visible peaks after the second peak in contrast to the poly-crystal.

Intriguingly, in the $g(r)$ for $\phi = 0.270$, the second peak disappears, while the first peak remains intact. This observation suggests that neighboring particles have a tendency to stay in close proximity, forming pairs.

References

[1] Caprini, L., Löwen, H.: Flocking without alignment interactions in attractive active brownian particles. Physical Review Letters **130**(14), 148202 (2023)

[2] Reis, P.M., Ingale, R.A., Shattuck, M.D.: Crystallization of a quasi-two-dimensional granular fluid. Phys. Rev. Lett. **96**, 258001 (2006) https://doi.org/10.1103/PhysRevLett.96.258001

[3] Carnahan, N.F., Starling, K.E.: Equation of state for nonattracting rigid spheres. J. Chem. Phys. **51**(2), 635–636 (1969)

[4] Chae, D.G., Ree, F.H., Ree, T.: Radial distribution functions and equation of state of the hard-disk fluid. J. Chem. Phys. **50**(4), 1581–1589 (1969)

[5] Chen, Y., Zhang, J.: High-energy velocity tails in uniformly heated granular materials. Phys. Rev. E **106**(5), 052903 (2022)

[6] Landau, L.D., Lifshitz, E.M., Kosevich, A.M., Pitaevskii, L.P.: Theory of Elasticity vol. 7. Elsevier, New York (1986)

[7] Schwager, T., Pöschel, T.: Coefficient of restitution for viscoelastic spheres: The effect of delayed recovery. Phys. Rev. E **78**(5), 051304 (2008)

[8] Scholz, C., Pöschel, T.: Velocity distribution of a homogeneously driven two-dimensional granular gas. Phys. Rev. Lett. **118**(19), 198003 (2017)

[9] Binder, K., Kob, W.: Glassy Materials and Disordered Solids: An Introduction to Their Statistical Mechanics. World scientific, Singapore (2011)
